# Air pollutants are negatively associated with vitamin D-synthesizing UVB radiation intensity on the ground

**DOI:** 10.1038/s41598-021-00980-6

**Published:** 2021-11-02

**Authors:** Abdur Rahman, Abdirashid Elmi

**Affiliations:** 1 Department of Food Science and Nutrition, College of Life Sciences, Kuwait University, Box 5969, 13060 Safat, Kuwait; 2 Department of Environmental Technology Management, College of Life Sciences, Kuwait University, P. O. Box 5969, 13060 Safat, Kuwait

**Keywords:** Environmental sciences, Environmental impact

## Abstract

Atmospheric levels of pollutants may reduce the UVB intensity at the earth’s surface, with a subsequent reduction in cutaneous vitamin D synthesis. We investigated the association of various pollutants with UVB intensity on the ground. Four-year data obtained from four weather stations from across Kuwait were analyzed by median regression. Pollutants that were negatively associated with UVB were [β (95% CI)]: benzene [− 2.61 (− 4.13, − 1.09)], ethyl-benzene [− 2.20 (− 3.15, − 1.25)], ozone [− 0.23 (− 0.28, − 0.17)], nitric oxide [− 0.11 (− 0.15, − 0.06)], sulfur dioxide [− 0.10 (− 0.17, − 0.04)] and particulate matter PM_10_ [− 0.002 (− 0.003, − 0.002)]. Pollutants that were negatively associated with the UVB/UVA ratio were [β (95% CI)]: benzene [− 15.57 (− 24.94, − 6.20)], nitric oxide [− 0.53 (− 0.81, − 0.25)], ozone [− 0.38 (− 0.70, − 0.06)], and total hydrocarbon [− 0.02 (− 0.04, − 0.01)]. Furthermore, benzene and nitric oxide levels were higher in the morning and evening hours, which are the times of most solar exposure in this region due to high temperature during midday. In addition to other known factors, attenuation of UVB by these pollutants may contribute to lower vitamin D levels in populations. In addition to direct public health hazard, these pollutants may contribute to the very high prevalence of VDD in this region.

## Introduction

Vitamin D deficiency (VDD) is wide spread in all age groups, both genders, all ethnicities, and all geographical locations^1,2^. Globally, an estimated one billion people are reported to have VDD^2^. The Gulf region of the Middle East has particularly high prevalence of VDD^3–6^. For example, high prevalence of VDD has been reported in adults from Saudi Arabia^7^, Bahrain^8^, Qatar^9^ and Jordan^10^. Prevalence of VDD is particularly high (> 90%) in adolescents. The prevalence of VDD in adolescents was 96% in Saudi Arabia^11^, 85–98% in India^12,13^, and around 97% in Korea^14^. In Kuwait, VDD and insufficiency has been reported to be of epidemic proportion in both adults and adolescent children, with only 20% of adults^15^ and 4% of adolescents^16^ having sufficient vitamin D levels.

Over 90% of vitamin D in the human body is synthesized in the skin when its precursor 7-dehydrocholesterol (7-DHC) is exposed to ultraviolet (UV) radiation from the sun^17^. It is thus generally assumed that populations in areas where there is ample sunshine throughout the year, would have adequate vitamin D levels^18^. Contrary to this popular assumption, there is a high prevalence of VDD and vitamin D insufficiency in the Middle East, an area with abundant sunshine^19,20^. Cultural practices, such as clothing that covers most of the body (particularly in women), sun-avoidance behavior due to hot climate most of the year, and the use of sun screen are some of the factors that may be responsible for this high prevalence of VDD. Staying indoor, either due to climatic conditions or occupation, is also a known risk factor for VDD^21^.

In addition to limited exposure to the sun, the efficiency of solar radiation to synthesize vitamin D in the skin may also be a factor in the high prevalence of VDD. This is evident from the fact that vitamin D levels in populations is on a decreasing trajectory despite the use of fortified foods^22,23^. Solar radiation in the UVB range (290–315 nm) photoisomerizes 7-DHC in the skin to precholecalciferol (previtamin D). This unstable metabolite is quickly converted in the skin, by thermal isomerization, to cholecalciferol (vitamin D_3_)^24^, which gets into the blood stream and is transported to various tissues by vitamin D binding protein (DBP). Contrary to the effect of UVB, longer wavelength UV radiation in the range of 320–400 nm (UVA) is known to degrade vitamin D in the skin^25^. Thus, the ratio of UVB/UVA will have significant consequences for the overall vitamin D status of the body. Blocking UVB in the air by a number of environmental contaminants, with minimal effect of UVA radiation would favor more vitamin D degradation in the skin and less vitamin D synthesis.

The intensity of the solar UVB radiation that reaches the earth surface is affected by atmospheric conditions through which it traverses. Both the path length (non-modifiable factors) and the composition of the atmosphere (modifiable factors) affect the intensity of solar UVB radiation at the earth’s surface. Solar zenith angle is the most important non-modifiable factor. The smaller the zenith angle, the shorter is the path length that the UVB traverses through, and the less are the chances of its absorption or deflection before it reaches the skin^26^. Zenith angle is affected by latitude, season and time of the day^17^. At higher altitude, the amount of UVB that reaches the surface is higher because of the less dense atmosphere and the shorter distance that the UVB has to traverse. For every 300 m increase in altitude, the amount of UVB that reaches the surface increases by 4%^26^.

The atmosphere through which the UVB traverses is not homogenous in different geographical locations and thus may have varying effects on UVB attenuation. UVB is more effectively attenuated by the atmosphere than UVA and visible light. Based on the Rayleigh scattering principles, the amount of scattering of electromagnetic radiation (in the UV range) by particles smaller than the wavelength of the electromagnetic radiation is inversely proportional to the fourth power of the wavelength^27^. Ozone (O_3_) is particularly important in attenuating UVB radiation^28^. O_3_ levels at any specific location can change by ~ 20% daily, depending on the level of pollution and the wind patterns^29^. In addition to O_3_, other airborne pollutants and clouds also attenuate UV radiation^28^, and thus may affect cutaneous vitamin D synthesis. Furthermore, atmospheric gases such as carbon monoxide (CO), nitrogen dioxide (NO_2_), and sulfur dioxide (SO_2_) can also modulate the intensity of solar radiation in the atmosphere, particularly the shorter wavelength radiation. A decrease of short wavelength solar radiation by 20–40% by these gases has been reported^30,31^.

Particulate matter (PM), of various sizes, is another environmental pollutant with potential UV attenuating potential. PM can absorb, scatter or diffuse solar radiation and thus would attenuate the UV radiation from reaching the earth’s surface. The UV-attenuating effect of PM depends on the density, composition and shape of the PM, which vary significantly with time and space^32^. A reduction of over 25% of UV radiation by PM has been reported^33^. Thus, the suspended PM in the air can indirectly affect the synthesis of vitamin D by attenuating the solar radiation reaching the skin.

Kuwait’s economy is largely based on the petro-chemical industry. This, together with the very heavy traffic load, may results in high levels of gaseous pollutants in the atmosphere. In addition, the desert climate of Kuwait favor the rising of dust, with more frequent dusty days. We, therefore, hypothesized that high concentrations of both particulate and gaseous pollutants in the atmosphere decrease the intensity of UVB photons reaching the skin, and this decrease in solar UVB output is contributing to the high prevalence of VDD in Kuwait. This hypothesis is based on several epidemiological studies that reported high prevalence of VDD in populations who lived in areas with high concentrations of both particulate and gaseous pollutants^34–40^. In this study, we investigated the association between the concentrations of pollutants in the air with the UVB radiation reaching the earth’s surface.

## Results

**Figure 1:**
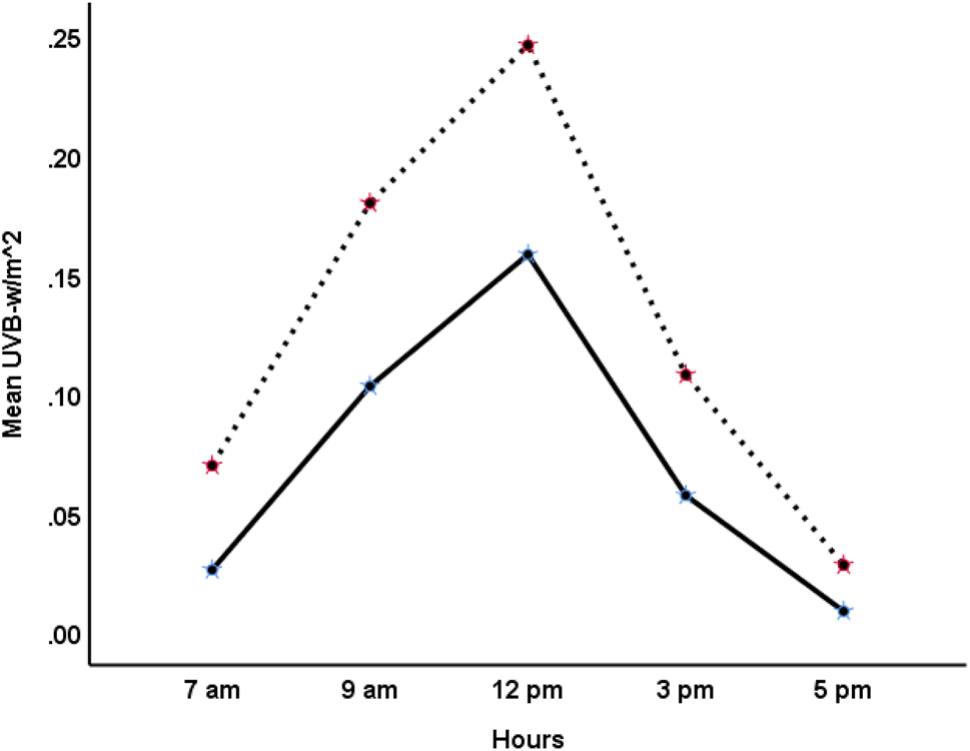
UVB intensity at earth’s surface at different hours in summer (March to September) and winter (October to February) months in Kuwait (Latitude range 28.30–30.00 N). Dotted lines represent summer months and solid line represents winter months.

**Figure 2:**
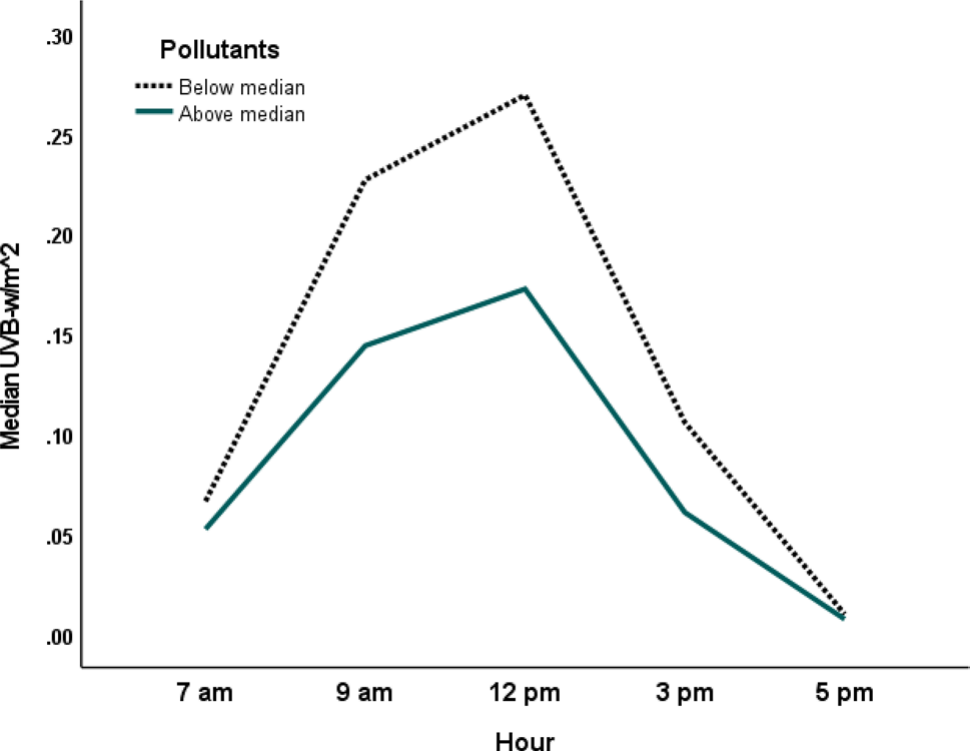
Attenuation of median UVB intensity at the ground by gaseous pollutants. A dummy variable was created by multiplying all the variables (included in Table 2) that were significantly associated with UVB (either positively or negatively). This dummy variable was then categorized into two groups (below median or above median) and median UVB was plotted for each group across different hours of the day. Dotted line represents UVB profile with pollutants level below the median and solid line represents UVB profile with pollutant level above the median. Repeated measure two-factor ANOVA showed that the two lines are significantly different (*p* < 0.001).

**Figure 3:**
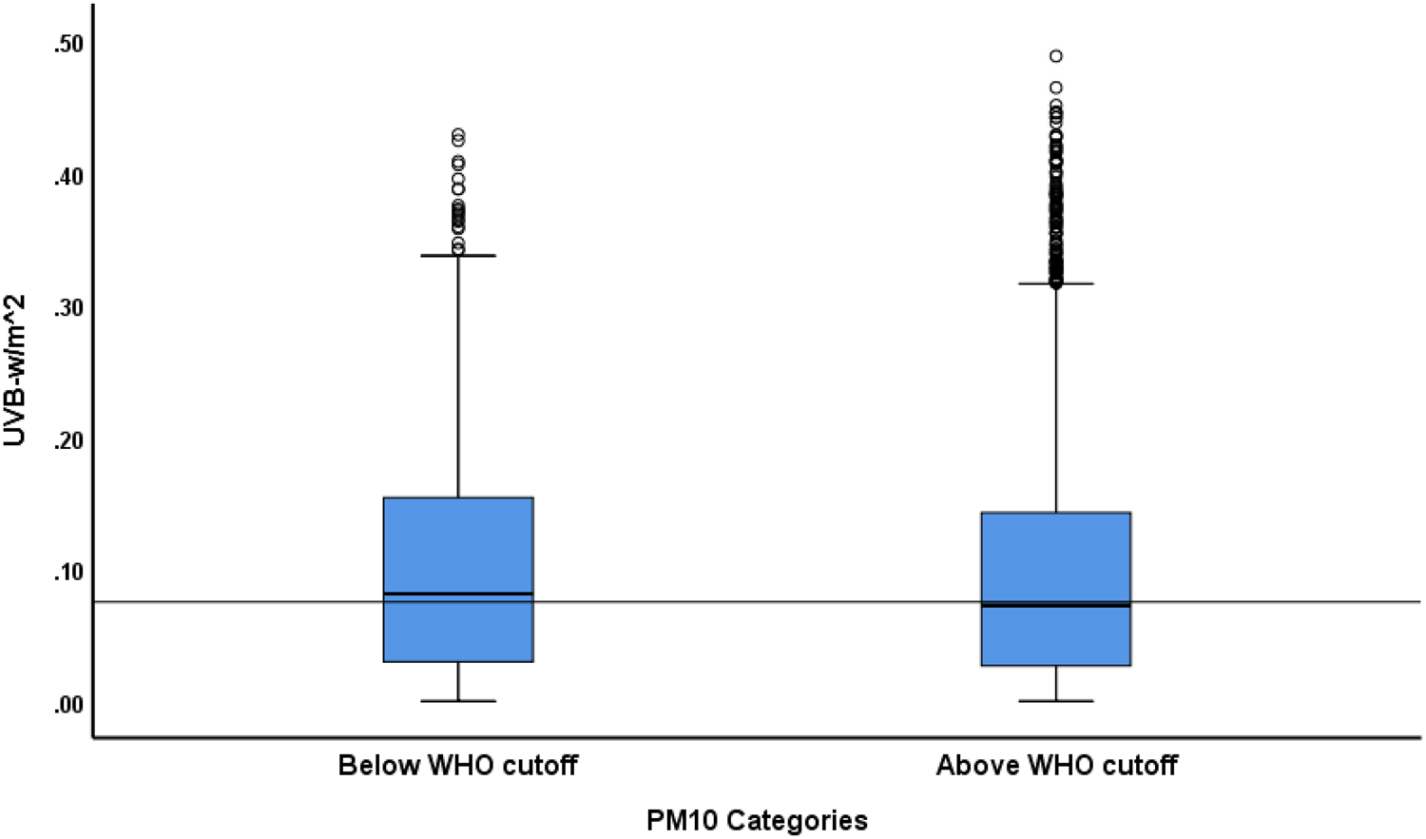
Attenuation of median UVB intensity at the ground by PM_10_. Median test and Kruskal–Wallis test, *p* < 0.001. The horizontal line represent overall median UVB (0.075 W/m^2^).

**Figure 4:**
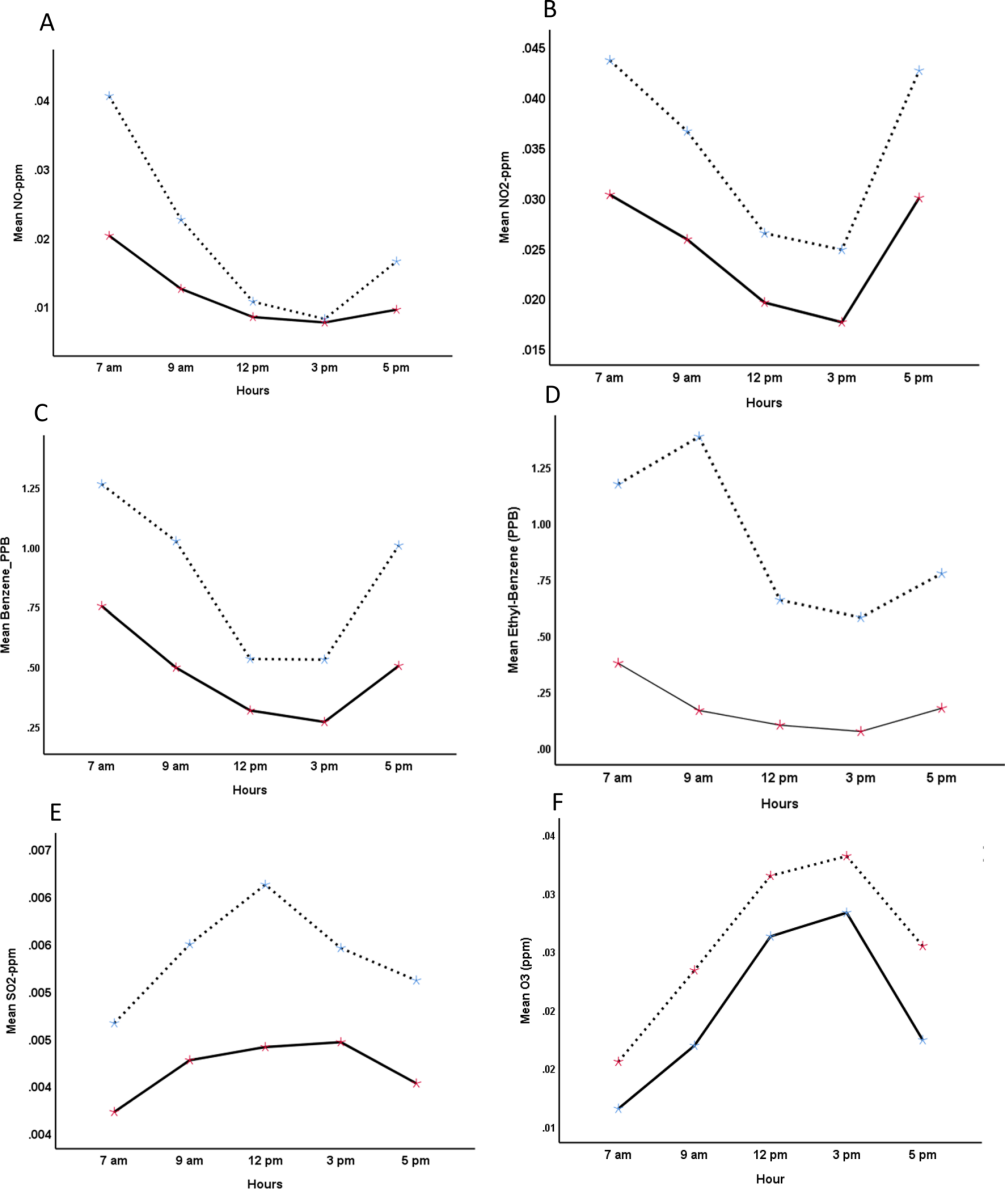
Distribution of important gaseous pollutants across various hours of the day in summer and winter months in Kuwait (Latitude range 28.30–30.00 N). Dotted line represent summer season (May to September) and solid line represent winter season (October to February). **A**: Nitric oxide (NO); **B**: Nitrogen dioxide (NO_2_); **C**: Benzene; **D**: Ethyl-benzene; **E**: Sulfur dioxide (SO_2_); **F**: Ozone (O_3_). Data are mean of the four stations for four years. Benzene and ethyl-benzene are in PPB, all other pollutants are in PPM.

Mean UVB intensity across different times of the day in summer (March to September) and winter (October to February) months are shown in Fig. 1. UVB intensity at earth’s surface was higher in summer months than winter months and peaked at noon (12 pm). The levels of various major gaseous and particulate (PM_10_) air pollutants in Kuwait are presented in Table 1. The PM_10_ content in the air was particularly high with the median PM_10_ 2.5-fold higher than the WHO standard. Of the 771 days for which data were analyzed, 613 days (80%) had PM_10_ above the WHO cutoff. Table 2 shows the median regression results on the association between various pollutants and UVB intensity. Major pollutants that were significantly (*p* < 0.01) negatively associated with UVB intensity were O_3_, SO_2_, NO, benzene, ethyl-benzene and PM_10_. Pollutants that were negatively associated (*p* < 0.01) with the UVB/UVA ratio were O_3_, NO, benzene and THC (Table 3). The UVB attenuating effects of the gaseous pollutants that were negatively associated with UVB intensity (Table 2) are shown in Fig. 2. Two-factor repeated measure ANOVA revealed that median UVB intensity at pollutants (combined) level below the median across different hours of the day was significantly higher (*p* < 0.001) compared to the median UVB at pollutants level above the median. Median (IQR) UVB intensity at the PM_10_ content below the WHO cutoff was significantly higher than when the PM_10_ content was above the WHO cutoff [0.08 (0.03–0.15) vs 0.07 (0.03–0.14); *p* < 0.001] (Fig. 3). Similar to the median, the distribution of UVB intensity between the two groups was significantly different (*p* < 0.001). Mean levels of O_3_ and SO_2_ NO, NO_2_, benzene and ethyl-benzene at different times in summer and winter months are shown in Fig. 4. As shown, mean levels of NO, NO_2_, benzene and ethyl-benzene were higher in the morning (7 and 9 am) and evening hours (3 and 5 pm) compared to the mid-day (12 pm) (Fig. 4A–D). On the other hand, mean levels of O_3_ and SO_2_ were higher during mid-day, compared to the morning or evening hours (Fig. 4E and F)**.**

**Table 1:** Descriptive statistics of various parameters.

Parameter	N	Mean	SD	Median	Min	Max	Percentiles			WHO air quality guidelines
							25	50	75	
SO_2_	16,222	0.01	0.01	0.003	0.00	0.27	0.003	0.003	0.005	0.0076 ppm^a^
NO	15,912	0.02	0.03	0.01	0.00	0.63	0.00	0.01	0.02	NA
NO_2_	15,912	0.03	0.02	0.02	0.00	0.32	0.01	0.02	0.04	0.106 ppm^a^
O_3_	17,190	0.02	0.01	0.02	0.00	0.18	0.01	0.02	0.03	0.05 ppm^b^
CO	17,131	0.71	0.51	0.60	0.00	6.16	0.35	0.60	0.96	9 ppm^b^
Benzene	2228	0.72	0.76	0.46	0.00	8.08	0.26	0.46	0.92	1.57 ppb (annual mean)^b^
Ethyl-Benzene	2228	0.63	1.22	0.13	0.00	22.13	0.00	0.13	0.69	NA
CH_4_	16,349	1.87	0.37	1.79	0.00	7.01	1.67	1.79	1.99	NA
THC	2261	2.23	0.43	2.18	1.12	8.38	2.02	2.18	2.33	NA
CO_2_	16,948	348.4	31.7	346.7	0.0	684.3	330.2	346.7	367.6	NA
Xylene	2228	2.55	4.57	1.19	0.00	65.22	0.43	1.19	2.92	NA
NH_3_	8304	0.03	0.03	0.02	0.00	0.51	0.01	0.02	0.03	NA
PM_10_	15,554	267	635	126	0	36,540	61	126	239	^a^50 ug/m^3^

PM10 is in µg/m^3^, Benzene, Ethyl-benzene and Xylene are in PPB, whereas all other gaseous pollutants are in PPM.

^a^Update of WHO air quality guidelines Michal Krzyzanowski & Aaron Cohen, Air Qual Atmos Health (2008) 1:7–13.

^b^Air quality guidelines for Europe; second edition.

*NA* not Available.

**Table 2:** Median regression showing the association of various environmental pollutants with UVB intensity at the earth’s surface.

Parameter	Absorption range (nm)	Coefficient (β)	95% CI of β	*P*-value	*R^2^
O_3_	200–315	− 0.23	− 0.28, − 0.17	< 0.001	0.77
SO_2_	190–230 280–320	− 0.10	− 0.17, − 0.04	< 0.01	
NO	260–320	− 0.11	− 0.15, − 0.06	< 0.001	
NO_2_	280–580	0.08	0.03, 0.14	< 0.01	
CO	< 280	0.01	0.01, 0.02	< 0.001	
CO_2_	230–300	< 0.001	< 0.001, < 0.001	< 0.01	
Benzene	< 280	− 2.61	− 4.13, − 1.09	< 0.01	
Ethyl-Benzene	< 290	− 2.20	− 3.15, − 1.25	< 0.001	
Xylene	< 280	0.53	0.15, 0.92	< 0.01	
PM_10_		− 0.002	− 0.003, − 0.002	< 0.001	

*Pseudo R^2^.

The full model included SO_2_, H_2_S, NO, NO_2_, CH_4_, NCH_4_, THC, O_3_, CO, CO_2_, NH_3_, Benzene, Toluene, Ethyl-benzene, Xylene, PM_10_, Total solar output, Temperature, Relative humidity, Hour of the day, Season, Station and Year. PM_10_ is in µg/m^3^; Benzene, Ethyl-benzene and Xylene are in PPB, other gaseous pollutants are in PPM. Parameters shown in the table were selected in the backward selection in the final model. Final model also included total solar output, relative humidity, year and season which are not shown in the table.

**Table 3:** Median regression showing the association of various environmental pollutants with UVB/UVA ratio.

Parameter	Coefficient (β)	95% CI of β	*P*-value	*R^2^
O_3_	− 0.38	− 0.70, − 0.06	0.02	0.07
NO	− 0.53	− 0.81, − 0.25	< 0.001	
NO_2_	0.93	0.62, 1.23	< 0.001	
CO	0.06	0.04, 0.08	< 0.001	
CO_2_	< 0.001	< 0.001, < 0.001	< 0.001	
Benzene	− 15.57	− 24.94, − 6.20	< 0.01	
Ethyl-Benzene	5.23	0.78, 9.69	0.02	
THC	− 0.02	− 0.04, − 0.01	< 0.01	
NH_3_	0.59	0.27, 0.91	< 0.001	
PM_10_	0.004	0.002, 0.006	< 0.001	

*Pseudo R^2^.

The full model included SO_2_, H_2_S, NO, NO_2_, CH_4_, NCH_4_, THC, O_3_, CO, CO_2_, NH_3_, Benzene, Toluene, Ethyl-benzene, MP-Xylene, O-Xylene, PM_10_, Total solar output, Temperature, Relative humidity, Hour of the day, Season, Station and Year. PM_10_ is in µg/m^3^; Benzene, Ethyl-benzene and Xylene are in PPB, other gaseous pollutants are in PPM. Parameters shown in the table were selected in the backward selection in the final model. Final model also included total solar output, relative humidity, year and season which are not shown in the table.

## Discussion

Our results are summarized as follows: (1) Levels of O_3_, SO_2_, NO, benzene, ethyl-benzene and PM_10_ were negatively associated with the UVB intensity at the earth’s surface. (2) Median UVB intensity at pollutants level below median was higher than the UVB intensity at pollutants level above the median. (3) The median PM_10_ level in the air was 2.5-fold higher than the WHO cutoffs of ambient air quality standards. (4) O_3_, NO, benzene and THC were negatively associated with the UVB/UVA ratio (5) Levels of NO, NO_2_, benzene and ethyl-benzene were higher in the morning and evening hours compared to the midday. The implications of these results are discussed below.

Among the gaseous pollutants studied, O_3_, SO_2_, NO, benzene, and ethyl-benzene were significantly negatively associated with UVB. Based on the strength of association (β), the UVB attenuating effects of these pollutants in decreasing order was: benzene > ethyl-benzene > O_3_ > NO > SO_2_. These results are theoretically plausible as all these pollutant have their absorption maxima in the UVB range. O_3_ absorbs radiation in the range of 200–315 nm; and thus would essentially blocks all the UV radiation < 290 nm, and attenuate the wavelengths > 290 nm^28^. O_3_ levels at any specific location can change by ~ 20% daily, depending on the level of pollution and the wind patterns^29^. SO_2_ absorbs radiation in the range of 280–320 nm, and its molecular absorption capacity is about 2.5-times that of O_3_. However, due to its smaller column thickness in the atmosphere, its total attenuating effect is smaller than O_3_^41^. Benzene absorbs maximally at wavelength below 280 nm, and ethyl-benzene’s absorption maxima is < 290 nm^42^. Similarly, NO absorbs maximally in the range of 260–320 nm. Attenuation of the short wavelength solar radiation by these pollutants in the troposphere has been well-known. A decrease of 20–40% of the solar radiation by atmospheric pollutants like CO, NO_2_, and SO_2_ has been reported^30,31^. High tropospheric O_3_ levels have been associated with reduced vitamin D synthesis in the skin and compromised bone health in postmenopausal women^39^. O_3_, CO, PM_10_, NO_2_, and SO_2_ are the major air pollutants with known adverse consequences to human health and the environment^43^. These pollutants are termed as “criteria pollutants” and are used to calculate ambient air quality globally.

Combustion of fossil fuel, either from automobiles or from petro-chemical industries (like flaring of unwanted gases), are the major sources of environmental NO and NO_2_^44^. These gases, in addition to their direct UVB attenuating effect, may also contribute to PM and O_3_ levels, both of which are negatively associated with UVB intensity at the earth’s surface. NO is the major source of tropospheric O_3_^45^. Approximately 10% of the O_3_ is in troposphere and 90% is in the stratosphere^44,46^. Whereas stratospheric O_3_ may be protective by blocking the dangerous radiations (complete blockage of radiation < 290 nm and partial blockage of 290–315 nm), the tropospheric O_3_ may have detrimental effects on vitamin D synthesis due to further attenuation of radiation in the range of 290–315 nm in the troposphere, which is required for vitamin D synthesis.

In addition to the gaseous pollutants, particulate pollutant PM_10_ was a significant negative predictor of solar UVB radiation. Although the effect size of PM_10_ in the median regression was very small (β = − 0.002), it was highly significant (*p* < 0.001). Similarly, when the UVB was stratified based on the whether the PM_10_ was below or above the WHO cutoff, the difference in the median UVB between the two categories of PM_10_ was small (0.081 vs 0.073), but highly significant. Although the coefficient is small, when we take into account the number of days per year with a PM_10_ content of above the WHO cutoff, the effect on blocking UVB could become significant. Based on the 4-year data analyzed in this study, the number of days with PM_10_ content above the WHO cutoff were approximately 80%. Thus the cumulative effects on the UVB content may be substantial. The UVB attenuating effect of PM depends on the density, composition, and shape of the particle^47^. Atmospheric PM can be either natural (dust) or anthropogenic, created from the combustion of fossil fuel in automobiles and industry^48^. In recent years the concentration of PM has increased by approximately 2% per year in both developed and developing countries^49^. The annual mean ambient PM of less than < 2.5 μm (PM_2.5_) in many countries exceeds the WHO safety level^50^. In addition to its direct UVB attenuating effect, PM can also indirectly affect cutaneous vitamin D synthesis by discouraging people from going outdoors.

Whereas, the UVB attenuating effects of “criteria pollutants” is reported, to the best of our knowledge, the UVB attenuating effects of benzene and ethyl-benzene have not been reported. In this study, benzene showed the strongest UVB attenuating effect among all pollutants studied, followed by ethyl-benzene. The major sources of atmospheric benzene is evaporation from petroleum products and exhaust emission from motor vehicles^51^. All these sources are abundant in Kuwait. Benzene, produced from petroleum products, is also used for the chemical synthesis of ethyl-benzene. Daily median air concentrations in the US have been reported as 0.16 ppb in remote areas, 0.47 ppb in rural areas and 1.8 ppb in urban/suburban areas^51^. Median daily benzene concentration in this study was 0.45 ppb with a maximum value of 8.08 ppb.

The effect of pollutants that are negatively associated with the UVB/UVA ratio (O_3_, NO, benzene and THC) has another dimension of relevance to the vitamin D status in population. Vitamin D is synthesized in the skin when the skin is exposed to UVB (290–315) nm. On the other hand longer wavelength UV (UVA) degrades vitamin D in the skin^25^. If any environmental pollutant selectively absorbs the UVB without any effect on the UVA, the UVB/UVA ratio will decrease. This situation will result in decreased synthesis and increased degradation of vitamin D in the skin. Thus exposure to sunlight in this situation would rather be detrimental than beneficial for vitamin D status.

The higher levels of pollutants like NO, benzene and ethyl-benzene in the morning and evening hours is an interesting observation and has implications for vitamin D status. Kuwait’s climate is such that the temperature remains high most days of the year, particularly during the summer months. Due to the high temperature, most people go out (for jobs, business and leisure) during the morning and evening hours and remain indoor during midday. In addition, indoor workers are only exposed to the sun during the morning and evening hours. VDD has been reported to be higher in indoor workers, compared to the outdoor workers^21^. Thus morning and evening hours are important times of exposure to sunlight. The intensity of UVB during these hours is lower compared to the midday (Fig. 2). The higher levels of these UVB blocking pollutants, together with lower intensity of the UVB during these hours may further compromise the vitamin D synthesizing ability of the skin. Seasonal variations in the concentrations of pollutants have been reported^52^. All the pollutants that were negatively associated with UVB intensity at the earth’s surface were higher in summer months compared to the winter months. Despite the high intensity of UVB in summer compared to winter season, high levels of these pollutants in summer may reduce the intensity of UVB, with adverse consequences for cutaneous vitamin D synthesis. This would have an additional impact on vitamin D synthesis apart from the usual sun-avoiding behavior of the population during summer months.

Consistent with these reported associations between environmental pollutants and the attenuation of UVB photons, atmospheric pollution has been associated with VDD by numerous epidemiological studies^34–39^. In Mexico city, where the levels of O_3_ and PM_2.5_ are high, 87% of children were reported to have VDD^36^. In India, toddlers who lived in an area of high atmospheric pollution had significantly lower level of vitamin D, compared to toddlers who lived in less polluted areas^34^. Low vitamin D levels were also reported from two highly polluted cities in Iran^38,40^.

With the exception of PM_10_, which was higher than the WHO standard, all gaseous pollutants that were negatively associated with UVB intensity are within the WHO limits of air quality standards. However, it is important to underline that the WHO ambient air quality guidelines are established for public health protection due to the toxicities of these pollutants, and may not necessarily explain much about the association between atmospheric pollutants and solar radiation intensity at the earth’s surface. Because of the strong UVB attenuating effects of some of these pollutants, further studies are needed to fully characterize the role, and the extent of contribution, of these pollutants in the VDD pandemic.

In this study, some environmental pollutants showed a statistically significant positive association with the UVB intensity. These included NO_2_, CO, CO_2_ and xylene. This could be explained by the photochemical formation (xylene and NO_2_ for example)^53^, and increased emission of these some during the daytime.

To our knowledge, this is the first study on the association between environmental pollutants and UVB intensity at the earth’s surface in the Gulf Cooperation Council (GCC) countries, a region with one of the highest rates of VDD. We utilized data that span over four years and represented both urban areas and rural arid areas. We identified benzene and its derivative ethyl-benzene as the strongest UVB-attenuating pollutants, which have not been studied before in this context. A limitation in this study is that we could not directly link the concentrations of these pollutants to vitamin D levels in population. However, the available data from both adolescents^16^ and adults^15^ show that VDD in Kuwait is widespread, which is in-line with our hypothesis.

## Conclusion

The data presented here demonstrate that benzene, ethyl-benzene O_3_, SO_2_, NO, and PM_10_ are negatively associated with the UVB intensity at the earth’s surface. The presence of higher concentration of these pollutants in the air, particularly during morning and evening hours, when most people get exposed to the sun, may contribute to the very high prevalence of VDD in Kuwait. Further studies are needed to establish the extent of interference of these pollutants with cutaneous vitamin D synthesis, and to establish standards that take into account the issue of vitamin D synthesis, in addition to the other adverse health effects of these pollutants.

## Methods

### Data acquisition

**Figure 5:**
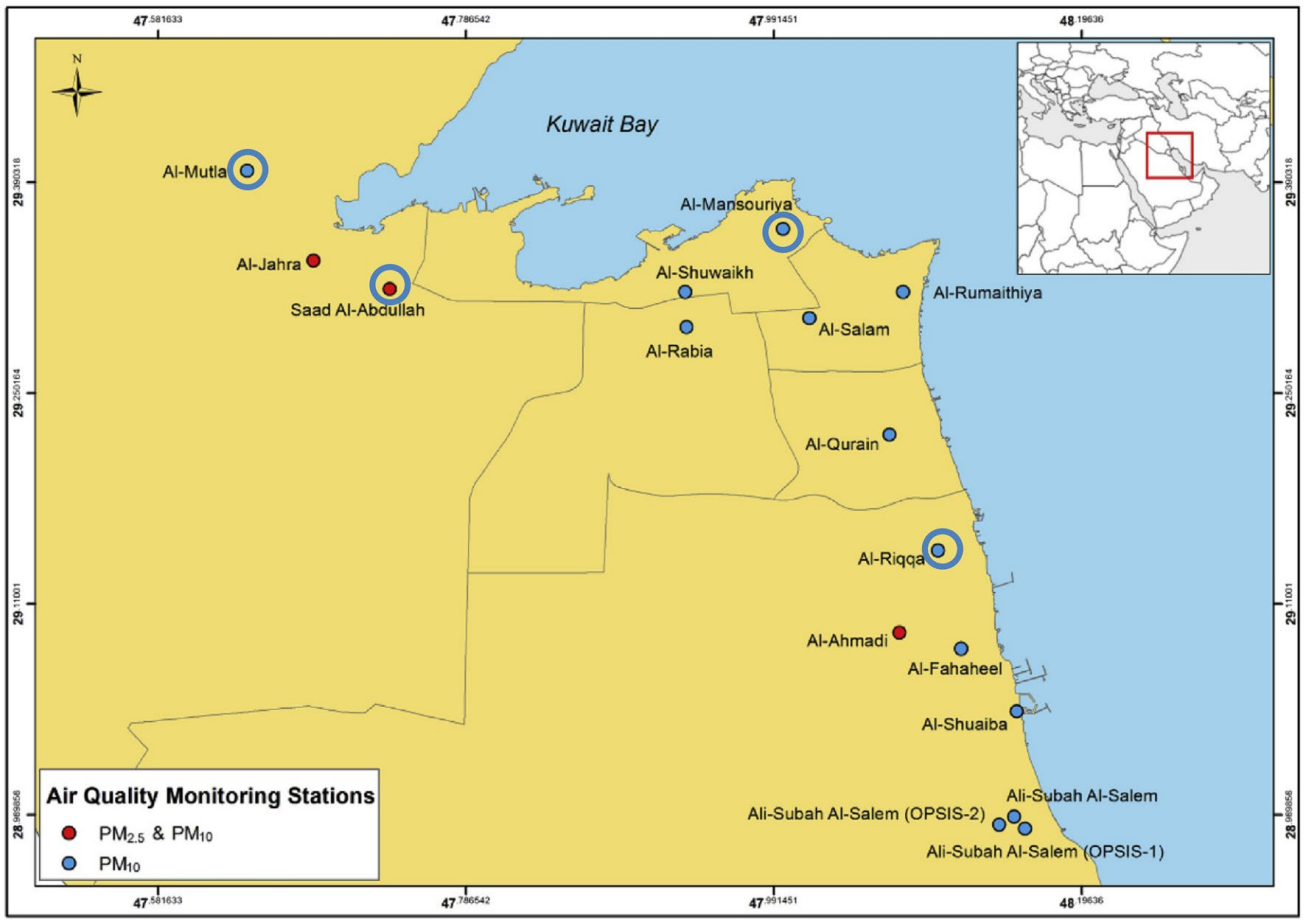
Location of the Kuwait EPA weather stations. The map is reproduced from Al-Hemoud et al.^54^ with permission from Elsevier.

Data on environmental factors were obtained from the Kuwait Environmental Protection Authority (EPA) monitoring stations. EPA has 18 weather stations across Kuwait (Fig. 5). In these stations, data are recorded on various parameters including environmental pollutants, both gaseous and particulate, total solar output, UVA and UVB radiation. Data from 15 stations were obtained for the years 2008 to 2011. Variables for which hourly data were available included sulfur dioxide (SO_2_), hydrogen sulfide (H_2_S), Nitric oxide (NO), nitrogen dioxide (NO_2_), methane (CH_4_), non-methane hydrocarbon (NCH_4_), total hydrocarbon (THC), O_3_, carbon monoxide (CO), carbon dioxide (CO_2_), ammonia (NH_3_), benzene, toluene, ethyl-benzene, xylene, PM_10_, total solar output, temperature, and relative humidity. As corresponding data on solar output was available from four stations, this study is based on analysis from four stations. These are Al-Mutla, Al-Mansoriya, Al-Riqqa and Saad Al-Abdullah (circled stations in Fig. 5). These centers represent both densely populated urban areas close to the cities and rural area on the outskirts of the city.

### Statistical analysis

The EPA weather stations collect data on hourly basis. We selected data for five time points i.e. 7 am, 9 am, 12 pm, 3 pm and 5 pm to represent times for the maximum light hours and times when people most likely go out during the summer and winter months. All the gaseous environmental pollutants were used as continuous variables in the analysis. PM_10_ data were used as continuous as well as categorized as binary variable based on the WHO cutoff of ambient air quality standard (50 µg/m^3^). Months were divided into two categories as summer (March to September) and winter (October to February) based on the average temperature during these months. As the UVB data were not normally distributed, we used median regression for assessing the association between UVB and various environmental pollutants. All models were adjusted for temperature, relative humidity, total solar output, season and time of the day. The first model included all the variables; variables that were not significantly associated were removed one by one using the backward selection. Total solar output was used in the models to remove the effect of clouds. The distribution of UVB across the two categories of PM_10_ was analyzed by Kruskal–Wallis test, whereas the medians were compared by the median test. For estimating the UVB attenuating effects of the gaseous pollutants, a dummy variable was created by multiplying all the variables (included in Table 2) that were significantly associated with UVB (either positively or negatively). This dummy variable was then converted into a binary variable (below median or above median) and median UVB was plotted for each group across different hours of the day. Two-factor repeated-measures ANOVA was used for main effect (pollutant levels) with hours as the repeated measure and median UVB intensity as the dependent variable. A p-value of 0.05 was used for statistical significance. Data were analyzed by SPSS (version 26).

## Data Availability

The datasets used and/or analyzed during the current study are available from the corresponding author on reasonable request.
